# A novel preliminary metabolomic panel for IHD diagnostics and pathogenesis

**DOI:** 10.1038/s41598-024-53215-9

**Published:** 2024-02-01

**Authors:** S. S. Markin, E. A. Ponomarenko, Yu. A. Romashova, T. O. Pleshakova, S. V. Ivanov, F. N. Bedretdinov, S. L. Konstantinov, A. A. Nizov, A. G. Koledinskii, A. I. Girivenko, K. M. Shestakova, P. A. Markin, N. E. Moskaleva, M. V. Kozhevnikova, Zh. Yu. Chefranova, S. A. Appolonova

**Affiliations:** 1 Institute of Biomedical Chemistry, Moscow, Russia 119121; 2 Belgorod Regional Clinical Hospital of St. Joseph, Belgorod, Russia 308007; 3 I.P. Pavlov Ryazan State Medical University, Ryazan, Russia 390026; 4 Peoples’ Friendship University of Russia, Moscow, Russia 117198; 5 Hospital Therapy No1, Department of the N.V. Sklifosovsky Institute of Clinical Medicine, I.M. Sechenov First Moscow Medical University (Sechenov University), Moscow, Russia 119435; 6 Laboratory of Pharmacokinetics and Metabolomic Analysis, Institute of Translational Medicine and Biotechnology, I.M. Sechenov First Moscow Medical University (Sechenov University), Moscow, Russia 119435; 7 World-Class Research Center Digital Biodesign and Personalized Healthcare, I.M. Sechenov First Moscow State Medical University (Sechenov University), Moscow, Russia 119435; 8 I.M. Sechenov First Moscow State Medical University (Sechenov University), Moscow, Russia 119435

**Keywords:** Biochemistry, Metabolomics, Cardiovascular diseases

## Abstract

Cardiovascular disease (CVD) represents one of the main causes of mortality worldwide and nearly a half of it is related to ischemic heart disease (IHD). The article represents a comprehensive study on the diagnostics of IHD through the targeted metabolomic profiling and machine learning techniques. A total of 112 subjects were enrolled in the study, consisting of 76 IHD patients and 36 non-CVD subjects. Metabolomic profiling was conducted, involving the quantitative analysis of 87 endogenous metabolites in plasma. A novel regression method of age-adjustment correction of metabolomics data was developed. We identified 36 significantly changed metabolites which included increased cystathionine and dimethylglycine and the decreased ADMA and arginine. Tryptophan catabolism pathways showed significant alterations with increased levels of serotonin, intermediates of the kynurenine pathway and decreased intermediates of indole pathway. Amino acid profiles indicated elevated branched-chain amino acids and increased amino acid ratios. Short-chain acylcarnitines were reduced, while long-chain acylcarnitines were elevated. Based on these metabolites data, machine learning algorithms: logistic regression, support vector machine, decision trees, random forest, and gradient boosting, were used for IHD diagnostic models. Random forest demonstrated the highest accuracy with an AUC of 0.98. The metabolites Norepinephrine; Xanthurenic acid; Anthranilic acid; Serotonin; C6-DC; C14-OH; C16; C16-OH; GSG; Phenylalanine and Methionine were found to be significant and may serve as a novel preliminary panel for IHD diagnostics. Further studies are needed to confirm these findings.

## Introduction

Cardiovascular disease (CVD) represents one of the leading causes of mortality worldwide^1^. Nearly half of the global cases of CVD are related to ischemic heart disease (IHD)^2^. IHD is a complex disorder, presumably resulting from metabolic dysfunction affected by different environmental and genetic impacts^3^. However, in many cases, IHD may be asymptomatic and occasionally remaining invisible until the onset of acute or irreversible stages of the disease^4–7^. To date, there are a number of clinically significant metabolic risk factors for the development of IHD including hypertension, type 2 diabetes mellitus and dyslipidemia. However, these factors do not always have a high degree of specificity and may not indicate the presence of pathology in a timely manner. Therefore, it is necessary to create more accurate and reliable approaches to predict the IHD and identify its hidden forms. It may be performed through modern in-depth diagnostic methods and comprehensive assessment of patient’s condition, particularly in the light of personalized medicine.

Metabolomic profiling represents a powerful tool for comprehensive analysis of small molecules in the considered biological fluids that may provide new biochemical insights into the IHD progression and characterize novel differences in the signaling pathways^8^. Unlike other OMICs technologies, metabolomic profile reflects the physiological state of the body at the current moment, therefore underlining the phenotypic changes in the body^9^. However, due to the nonlinearity of metabolomic data, as well as high interindividual variability, analysis of the results of metabolomic profiling requires the use of progressive bioinformatics methods of analysis.

In recent years, artificial intelligence approaches, in particular machine learning (ML) methods, have attracted special attention in metabolomics^10–12^. ML methods are mathematical functions applied in the optimization process using input and output data. In other words, the ML model makes a prediction based on associations between the values of its constituent features. The use of supervised ML classification methods makes it possible to build predictive models based on the training data set, which allows further stratifying patients with respect to the considered diseases. For today, there is a number of works focused on the development of ML-based diagnostic models. These models may be used in future for reliable patient stratification and timely diagnosis of IHD.

Thus, the aim of the study was to identify the key metabolites and metabolic pathways of IHD and to create on its basis the pilot ML-model for IHD diagnostics and pathogenesis.

## Materials and methods

### Study design

Inclusion and exclusion criteria of the study are presented in the Table 1.

**Table 1:** Inclusion and exclusion criteria.

Inclusion criteria	Men and women aged 18 years and older Angina pectoris functional class III according to Canadian Cardiovascular Society classification Availability of signed and dated informed consent of the patient to participate in the study
Exclusion criteria	Angina pectoris functional class I, II or IV Type 1 Diabetes mellitus Acetaminophen, all vitamins, minerals, amino acids, dietary supplements, including sports drinks and energy drinks, creatinine, alpha-ketoglutarate, malic acid, citric acid, maleic acid, orotic acid consumption during 4 days before blood sampling. Sweeteners (aspartame, among others), monosodium glutamate and alcohol intake 24 h before blood sampling Any other diseases or conditions that, in the opinion of the investigator, may distort the results of the study and limit the patient’s participation in the study

In total 137 patients with IHD were screened, of whom 84 patients with IHD were initially enrolled in the study. 53 patients had exclusion criteria, the most common of them were angina pectoris functional class I (n = 12), II (n = 25) or IV (n = 11), Type 1 DM (n = 5).

62 non-CVD subjects were screened, of whom 43 subjects were enrolled in the study, other 19 subjects had IHD.

5 patients from IHD group and 4 subjects from non-CVD group were excluded from the study due to diet violation (energy drinks consumption), 3 patients from IHD group and 3 subjects from non-CVD group were excluded due to alcohol consumption.

Patients diagnosed with IHD had angina pectoris functional class III according to Canadian Cardiovascular Society classification and a combined dyslipidemia characterized by elevated triglycerides and decreased HDL cholesterol^13^. IHD patients used organic nitrates, β-blockers, calcium channel blockers, ACE inhibitors, ARBs and statins.

The non-CVD group consisted of adults without any clinical or laboratory signs of cardio-vascular pathology and the risk factors of IHD.

Information on demographics, medical history, biochemical analysis and patient’s treatment was provided from the hospital database.

### Ethical considerations

All conducted experiments were approved by the Ethics Committee of Belgorod Regional Clinical Hospital of St. Joseph, Belgorod, Russia (protocol #10 from 16 of November, 2015) in conformity with the ethical principles for medical research involving humans stated in the Declaration of Helsinki. Written informed consent was signed by all the participants before the beginning of the study.

### Anthropometric evaluation

The anthropometric evaluation included measurements of weight, height and body mass index (BMI).

### Biochemical analysis

Whole blood samples were collected into ethylenediaminetetraacetic acid (EDTA) tubes, immediately centrifuged (2000 rpm, 4 °C) during 20 min to receive plasma and stored at − 80 °C. Following biochemical evaluation of the samples included measurements of total cholesterol, triglycerides, high density lipoproteins (HDL), alanine aminotransferase (ALT), aspartate aminotransferase (AST), creatinine, glucose, fibrinogen, international normalised ratio (INR), activated partial thromboplastin time (APTT). Extra plasma aliquots were utilized for the metabolic analysis in the Laboratory of pharmacokinetics and metabolome analysis.

### Chemicals and reagents

Standard solutions for metabolomic profiling, methanol, formic acid, bovine serum albumin (BSA) were received from Sigma-Aldrich (USA). Acetonitrile was purchased from Chromasolv® (Sigma-Aldrich Chemie GmbH, Buchs, Switzerland). Ultrapure water was received through the Millipore Milli-Q purification system (Millipore Corporation, Billerica, MA). Isotopically-labeled standard solutions for metabolic profiling Amino Acids and Acylcarnitines were received from MassChrom Non Derivatized 57000 Kit (Chromsystems, Germany), whereas isotope-labeled standard solutions for tryptophan catabolites profiling were from Toronto Research Chemicals (USA).

### Metabolomic profiling

Targeted metabolomic profiling of the samples was performed in accordance to the method presented previously^14^ and included quantitative analysis of 87 endogenous metabolites in the patient’s plasma. Briefly, sample preparation of amino acids, intermediates of Arginine and Methionine metabolism consisted of protein precipitation with following instrumental analysis on Waters TQ-S-micro triple quadrupole mass spectrometer (Waters Corp, Milford, CT, USA). Preparation of samples for acylcarnitine and tryptophan catabolite profiling consisted of liquid–liquid extraction followed by LC–MS/MS analysis. The applied methods were validated in accordance with the guidelines for bioanalytical method validation and included assessment of selectivity, linearity, precision and accuracy, recovery, matrix effect, and stability of the methods.

### Statistical analysis

To exclude the influence of age on the results of the metabolomic profiling we performed its correction using the regression analysis modeling (Python)^15^. The algorithm of the adjustment was following:

- Select the group of non-CVD subjects and divide it into 5-year stratum.
- Calculate median values of each metabolite in each stratum.
- Based on the selected median values in separated stratums build linear regression model and calculate regression coefficients.
- According to the received regression results calculate delta in concentration changes associated with age.
- Extract the calculated delta from each absolute concentration.

All further statistical analyses for characterization of biochemical and metabolic profiling measurements were performed using the Python Stats package. Variable distribution was assessed using the Shapiro–Wilk test. According to the variable distribution, the analysis of variance was performed using parametric student t-test and ANOVA test or using non-parametric Mann–Whitney U test. The p-value less than 0.05 was considered as significant.

### Development of the diagnostic model using machine learning algorithms

Further, to elucidate the best diagnostic model of IHD we applied and trained five machine learning algorithms, including: logistic regression (LR)^16^, support vector machine (SVM)^17^, decision trees (DT)^18^, random forest (RF)^19^ and gradient boosting (GB)^20^. LR and SVM with linear kernel relate to the class of linear classifiers that serves for categorizing a set of data point into a discrete class according to the linear combination of its explanatory variables. At the same time, DT, RF and GB are related to the non-linear class of algorithms. In DT classification procedure starts at the tree’s root node, where it assesses the attribute specified by this node, then moving down the tree branch corresponding to the attribute's value, as shown in the above figure. This procedure is repeated for each subset in a recursive partitioning manner. The RF and GB models are ensemble ML methods based on decision trees algorithms. In RF the predictions are performed by calculation the average of multiple trees’ output. As the number of trees increases, so does the precision of the output^21^. Contrary, GB algorithm represents an additive model which determines the impact of a poor learner by means of the gradient descent optimization. Thus, in this case the impact of each tree is assessed through the decrease of the overall error of the strong learner^22^.

Assessment of ML algorithms performance was performed using quality assessment metrics. For this purpose, we calculated parameters of confusion matrix, including true positive (TP), false positive (FP), true negative (TN), and false negative (FN) for actual and predicted data, based on which we further evaluated following metrics: area under the curve (AUC), accuracy, f1-score and recall.

## Results

**Figure 1:**
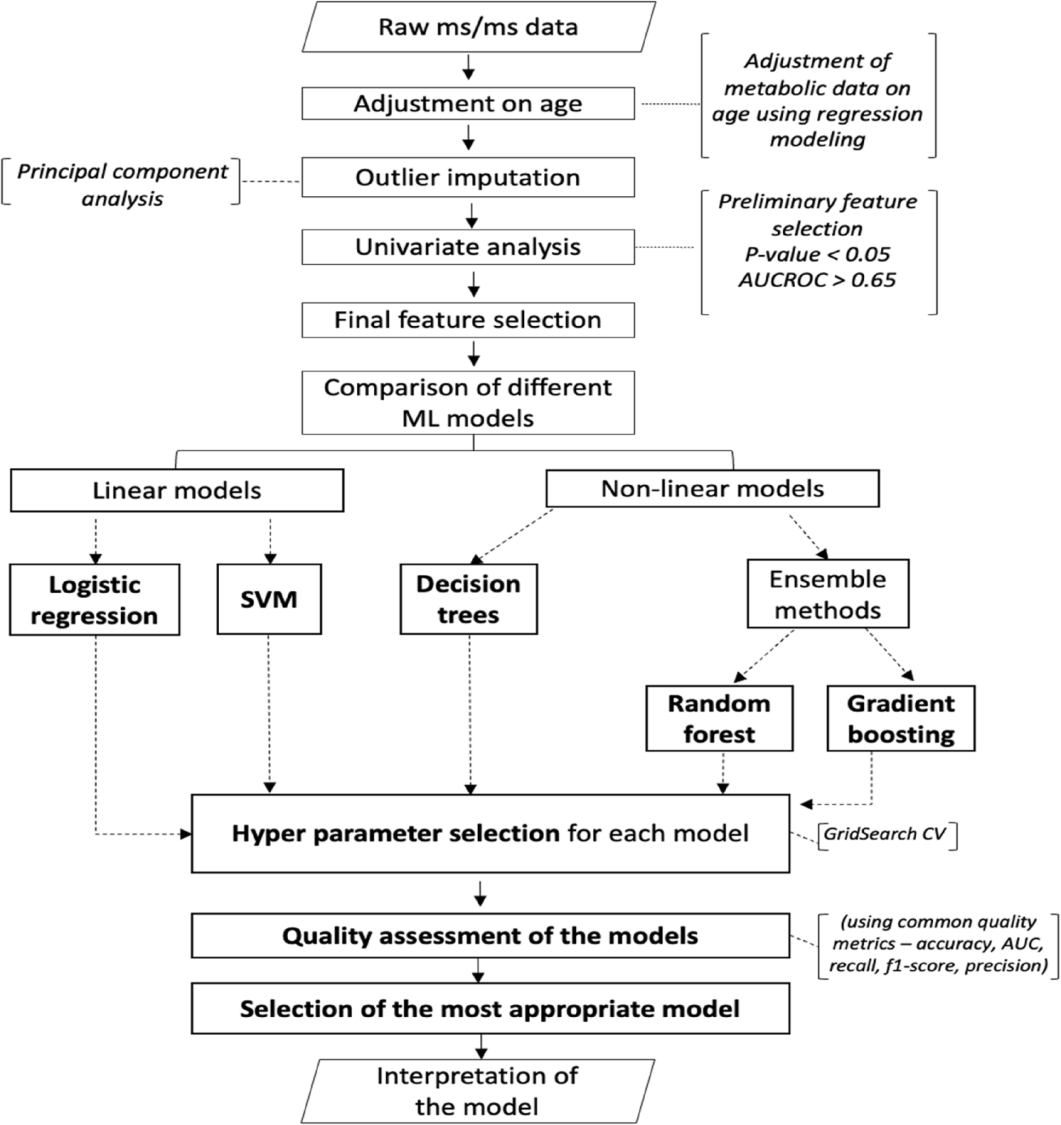
Flowchart on the processing of the metabolic profiles of non-CVD patients and patients with IHD.

The presented study was conducted in accordance with the flowchart presented in Fig. 1.

### Baseline characteristics of the IHD patients and non-CVD subjects

Among the considered subjects, the IHD patients were older than subjects from the non-CVD group and were characterized by higher body weight and higher BMI values.

The lipid analysis showed that total cholesterol was in normal range, but high triglycerides and low HDL cholesterol were observed that are characteristics of combined dyslipidemia.

The measurements of ALT, AST and glucose were in normal range. The creatinine level was increased in IHD group vs the non-CVD nevertheless it was in normal range. The coagulogram showed the normal range of fibrinogen, INR and APTT in both groups.

More information concerning the characteristics of patients is represented in Table 2.

**Table 2:** Baseline characteristics of the IHD patients and non-CVD subjects.

Variable	Non-CVD group (n = 36)	IHD group (n = 76)	p-value
Gender, m/f (%)	29/7 (80/20)	65/11 (86/14)	< 0.04
Age (years)	34 [26–44]	64 [58–71]	< 0.0001
Height (m)	1.67 [1.62–1.72]	1.69 [1.64–1.75]	0.21
Weight (kg)	69.0 [59.0–81.0]	84.4 [70.0–89.0]	< 0.0001
BMI (kg/m^2^)	24.6 [20.7–27.4]	29.5 [25.6–31.63]	< 0.0001
Heart rate/min	62.15 ± 10.83	65.81 ± 9.24	0.51
Systolic BP (mm Hg)	119.5 ± 5.6	120.3 ± 6.9	0.82
Diastolic BP (mm Hg)	75.9 ± 6.2	74.7 ± 5.9	0.78
Total cholesterol (mmol/L)	5.27 [4.8–5.9]	5.55 [4.50–6.20]	0.48
HDL cholesterol (mmol/L)	1.51 [1.22–1.78]	1.08 [0.89–1.19]	< 0.001
LDL cholesterol (mmol/L)	3.3 [2.7–4.0]	3.3 [2.9–4.1]	0.61
VLDL cholesterol (mmol/L)	0.5 [0.3–0.7]	1.0 [0.9–1.2]	0.01
Triglycerides (mmol/L)	1.10 [0.76–1.28]	1.73 [0.96–2.18]	< 0.01
ALT (u/L)	23.0 [20.0–24.0]	29.0 [20.0–34.5]	0.31
AST (u/L)	25.0 [23.0–27.0]	36.0 [20.0–36.0]	0.66
Creatinine (µmol/L)	89.1 [79.4–97.2]	100.5 [85.5–111.6]	0.01
Glucose (mmol/L)	4.88 [4.59–5.30]	5.65 [4.70–5.88]	< 0.01
Fibrinogen (g/L)	2.25 [2.0–2.30]]	3.28 [2.53–3.74]	< 0.05
INR	1.09 [1.05–1.14]	1.23 [1.11–1.28]	< 0.05
APTT (s)	27.2 [25.5–28.6]	31.5 [27.0–34.8]	0.19

General and biochemical characteristics of the participants including n (%) or median and interquartile range [Q1; Q3] in the considered groups and corresponding p-values, characterizing statistically significant differences between groups.

### Univariate analysis of the concentration levels of the metabolites

Due to the relationship between the metabolomic profiling and age, as well as the large difference in the age characteristics of the proposed groups of patients, we adjusted the results of the metabolomic profile using regression models. Conversion factors are presented in the Table S1. Further, identification of the metabolites that significantly altered among the considered groups of patients was performed using parametric and non-parametric comparison tests. Table 3 summarizes information on the significantly changed metabolites including class of the metabolite, direction of change and adjusted p-value, AUC score and Younden index.

**Table 3:** Significant metabolites with the consequent direction of their concentration change between the non-CVD and IHD groups. Metabolites with p-value < 0.05 and AUC score > 0.65 were selected as meaningful.

Metabolite	p-value	Direction	AUC score	Younden index
Acylcarnitine profiling				
Hydroxyhexadecanoylcarnitine	< 0.0001	Increased	0.84	1.69
Hydroxyhexadecenoylcarnitine	< 0.05	Increased	0.64	1.68
Hydroxytetradecanoylcarnitine	< 0.001	Increased	0.73	1.68
Adipoylcarnitine	< 0.01	Increased	0.66	1.68
Glutarylcarnitne	< 0.001	Decreased	0.70	1.71
Propionylcarnitine	< 0.05	Decreased	0.63	1.78
Carnitine	< 0.0001	Decreased	0.75	1.98
Tryptophan metabolism pathway				
Tryptophan	< 0.05	Decreased	0.63	1.86
Serotonin	< 0.0001	Increased	0.75	1.93
Indole-3-propionic acid	< 0.001	Decreased	0.72	1.84
Indole-3-butyric acid	< 0.01	Decreased	0.68	1.69
Indole-3-carboxaldehyde	< 0.05	Decreased	0.62	1.70
Kynurenic acid	< 0.01	Increased	0.67	1.69
Anthranilic acid	< 0.001	Increased	0.73	1.68
Xanthurenic acid	< 0.05	Increased	0.62	1.69
NO/urea cycle				
Asymmetric dimethylarginine	< 0.01	Decreased	0.70	1.83
Arginine	< 0.01	Decreased	0.68	1.91
Citrulline	< 0.01	Decreased	0.67	1.92
Amino acid profiling				
Isoleucine	< 0.01	Increased	0.68	1.95
Threonine	< 0.0001	Decreased	0.82	1.99
Histidine	< 0.0001	Decreased	0.80	1.98
Phenylalanine	< 0.0001	Increased	0.79	1.99
Proline	< 0.0001	Decreased	0.74	1.98
Lysine	< 0.001	Decreased	0.72	1.96
Glycine	< 0.01	Decreased	0.68	1.88
Leucine	< 0.05	Increased	0.65	1.95
Aspartic acid	< 0.05	Decreased	0.62	1.69
Asparagine	< 0.01	Decreased	0.67	1.83
3-Aminoisobutyric acid	< 0.05	Increased	0.64	1.96
Tyrosine	< 0.05	Decreased	0.62	1.82
Cystathionine cycle				
Cystathionine	< 0.05	Up	0.64	1.73
Methionine sulfoxide	< 0.01	Decreased	0.68	1.80
Norepinephrine	< 0.0001	Decreased	0.76	1.69
Dimethylglycine	< 0.01	Increased	0.66	1.93
Significantly changed ratios				
Fischer ratio	< 0.05	Increased	0.65	1.92
GSG ratio	< 0.05	Increased	0.63	1.92

The above given results showed that:

- Cystathionine and dimethylglycine (DMG) were significantly increased in IHD patients. At the same time, NO/urea cycle intermediates (ADMA, arginine and citrulline), as well as methionine sulfoxide and norepinephrine were significantly decreased.
- Intermediates of tryptophan catabolism including serotonin, anthranilic acid, kynurenic acid and xanthurenic acid were significantly increased, whereas tryptophan, indole-3-carboxaldehyde, indole-3-propionic acid and indole-3-butyric acid significantly decreased.
- Amino acids phenylalanine, branched-chain amino acids (BCAA) (isoleucine, leucine) and 3-aminoisobutyric acid were significantly elevated in the IHD patients. At the same time, aspartic acid, asparagine, tyrosine, glycine, lysine, proline, histidine and threonine were significantly decreased. Moreover, Fisher ratio ((Val + Ile + Leu)/(Phe + Tyr)) and GSG ratio (Glu/(Ser + Gly)) were significantly elevated in the IHD patients.
- Levels of short chain acylcarnitines, including C0, C3 and C5-DC were significantly decreased in IHD patients, whereas C6-DC and hydroxylated long-chain acylcarnitines (C14-OH, C16-1-OH and C16-OH) were significantly increased.

**Figure 2:**
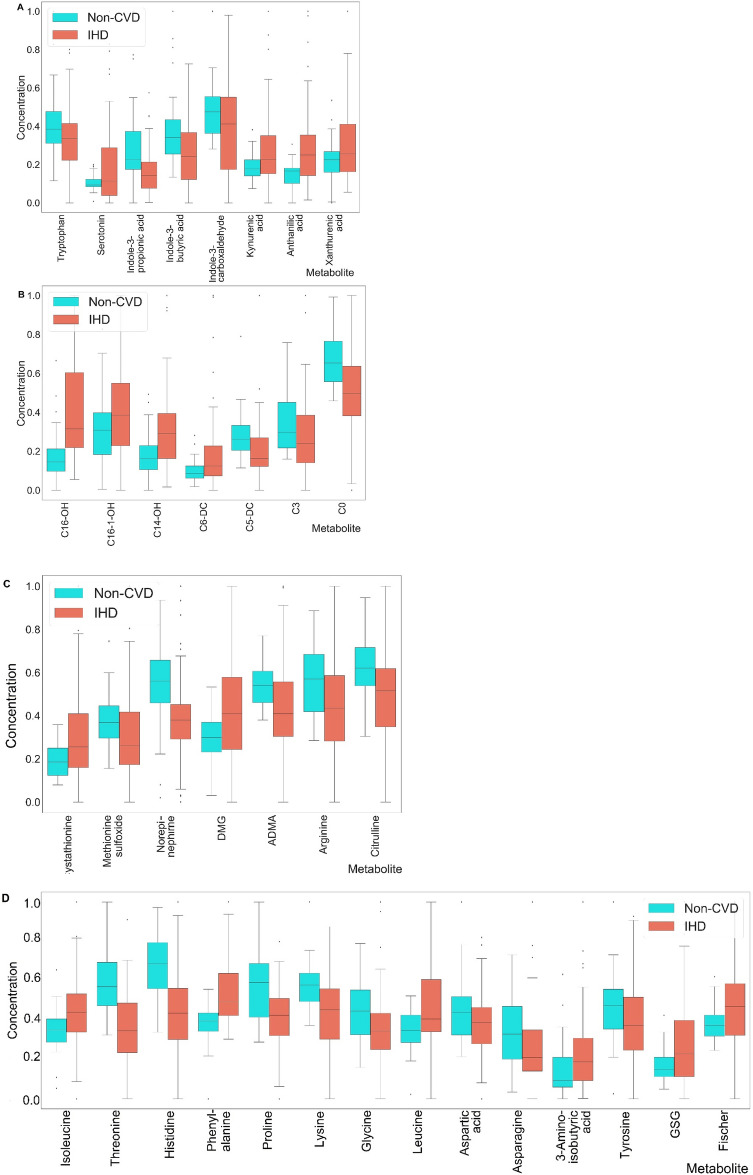
The box-plots of the significantly changed metabolites of: (**A**) tryptophan catabolism pathway; (**B**) acylcarnitine profiling; (**C**) cystathionine, betaine and arginine profiling; (**D**) amino acid profiling. The statistically significant metabolites were selected using parametric Student t-test (for normally distributed values) or equivalent non-parametric Mann–Whitney test (p < 0.05).

Graphical interpretation of results after min–max normalization are presented in Fig. 2A–D (A—Tryptophan metabolism intermediates; B—acylcarnitine profiling metabolites; C—cystathionine, betaine and arginine pathway intermediates; D—amino acid profiling metabolites).

**Figure 3:**
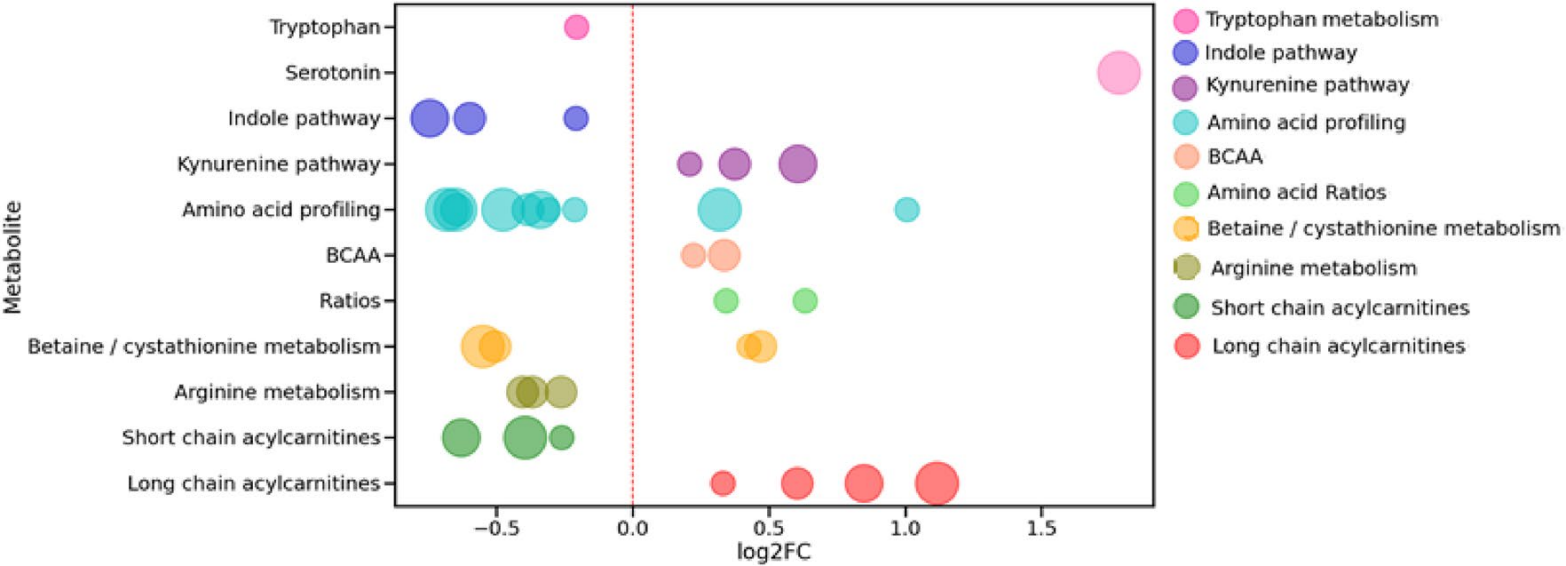
Bubble plot of the significantly changed metabolic pathways for IHD patients compared to the non-CVD subjects. Each bubble represents the identified significantly changed metabolite, whereas its color indicates the involvement in the corresponding metabolic pathway, and its size corresponds to the magnitude of its p-value (the size of the bubble positively correlates with the p-value magnitude). The position of each bubble characterizes the value of log2FC between its concentration in IHD patients and subjects without CVD.

On the basis of association of significantly changed metabolites are known metabolic pathways a bubble plot was created (Fig. 3).

### Development of the ML models based on the results of the metabolomic profiling

First of all, for a general overview of the received data and outlier exclusion, we performed a principal component analysis (PCA) (Supplementary Material Fig. S1). It has revealed that groups may be partly separated from each other.

**Figure 4:**
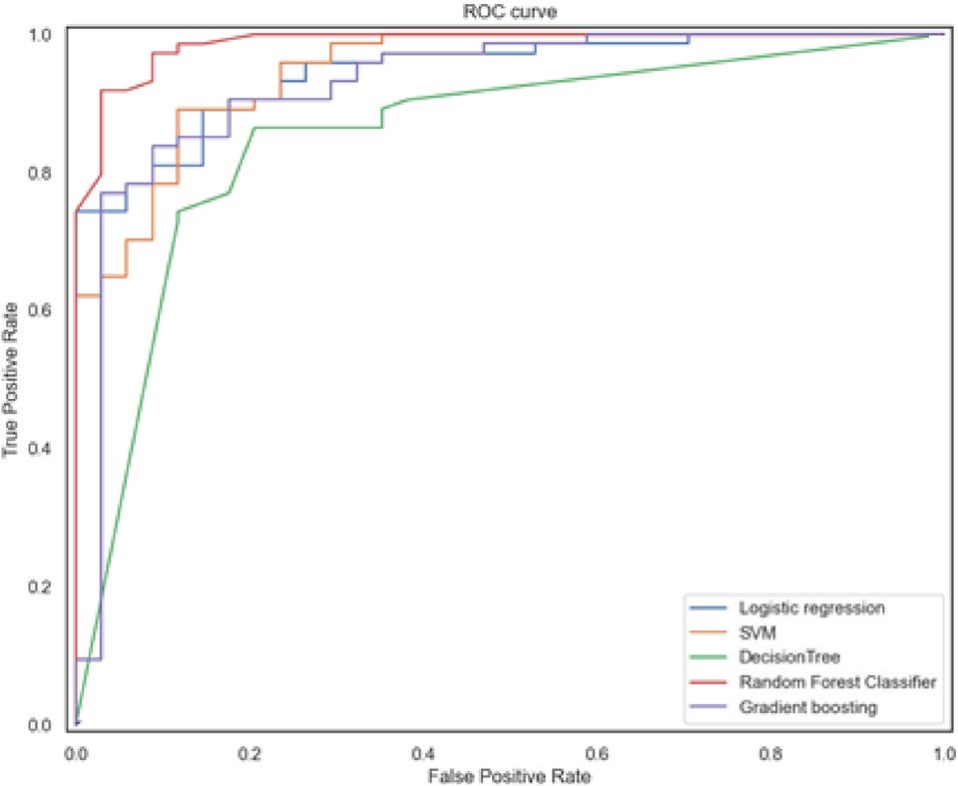
AUC ROC analysis of the applied machine learning methods. Random Forest model represents the best diagnostic accuracy.

Further, to identify the most appropriate prediction machine learning (ML) based model, we compared different supervised ML algorithms, including LR, SVM, DT, RF, and GB (Table 4). Each model was built based on the metabolic biomarker features using a cross-validated Python Gridsearch approach to identify of the best hyperparameters. The tuned hyperparameters of the ML models are presented in Supplementary Table S2. To determine the most precise diagnostic model of IHD there were applied common quality assessment metrics: sensitivity, specificity, AUC and confusion matrix together with the cross-validation method in splitting the working dataset. Figure 4 represents the AUC ROC of the developed ML models. The RF algorithm showed the best quality compared to the other used methods.

**Table 4:** The list of the applied ML-algorithms and corresponded quality metrics (confusion matrix, sensitivity, specificity, accuracy, AUC ROC).

ML algorithms	Confusion matrix (TP, FP, FN, TN)		Sensitivity	Specificity	Accuracy	AUC ROC
Logistic regression	28	8	0.82	0.89	0.87	0.94
	6	66				
SVM	26	4	0.76	0.96	0.89	0.96
	8	70				
Decision trees	24	10	0.71	0.86	0.82	0.86
	10	64				
Random forest	26	0	0.76	1.00	0.93	0.98
	8	74				
Gradient boosting	23	4	0.67	0.95	0.86	0.93
	11	70

## Discussion

### Univariate changes of the metabolomic profiling in IHD patients in comparison with non-CVD subjects

To the best of our knowledge it is the first complex investigation of the metabolomic profile and ML model for IHD, which comprises the main pathways of its diagnostics and pathogenesis.

In accordance with the received results, methionine metabolism was significantly affected during the CVD progression, showing increased levels of cystathionine. Elevated cystathionine plasma levels are related to endothelial dysfunction characterized by reduced nitric oxide-mediated vasodilation of arteries, therefore causing atherosclerotic lesions^23^. Besides this, cystathionine also affects glutathione production, which causes oxidative stress, thus inactivating nitric oxide production.

On the other hand, there was a significant decrease in the concentration of methionine sulfoxide—methionine derivative. Similarly to cystathionine, increased levels of methionine sulfoxide possess oxidative stress in the body^24,25^.

DMG and glycine were found to be significantly decreased in the IHD group. The glycine is known as a biomarker of cardiovascular dysregulation^26^, and its decreased level in IHD patients was expected, but a significant decrease in DMG level was firstly found and was unexpected.

Short-chain acylcarnitines (C0, C3, C5-DC) were significantly decreased in IHD group vs non-CVD group. Plasma concentrations of these acylcarnitines are known to reflect the gut microbiota and amino acid metabolism. C3 and C5 acylcarnitines are known as direct products of BCAA catabolism^27^, BCAA (leucine and isoleucine) were significantly increased in the IHD group. Hydroxylated long-chain acylcarnitines (C14-OH, C16-1-OH and C16-OH) were significantly increased in the IHD group. In general, long-chain acylcarnitines are known as markers of cardiovascular disorders^28–30^. Dysregulation in long chain acylcarnitines is usually associated with mitochondrial fatty acid oxidation disorders. However, little is known about the function of hydroxylated acylcarnitines in the IHD pathogenesis.

Arginine is the primary precursor for nitric oxide (NO) production in the vascular endothelium. Therefore, decreased arginine levels and its primary metabolite—ADMA—in IHD patients may indicate the lack of NO production. Additionally, there were also found significantly decreased levels of citrulline—endogenous metabolite, that is connected to arginine via the urea cycle being its end-product.

Intermediates of aspartate metabolism—aspartate and asparagine were significantly decreased in IHD patients. Asparagine is known as a glucogenic amino acid. Previously, asparagine was shown to be associated with high risks of cardiometabolic disease^31^. Along the aspartate metabolic pathway, asparagine is converted to aspartate and further through transamination to glutamate. Glutamate, glycine (also significantly decreased in IHD group) and cysteine represent the basis for the formation of tripeptide glutathione, which was also decreased. Glutathione is one of the major antioxidant in the body and its decreased level plays the main role in the atheroprogression in the smooth muscle and the endothelial cells^32^.

Amino acid ratios (Fisher and GSG) were increased in IHD group. The Fisher ratio represents the sum of BCAA divided by the sum of aromatic amino acids (Tyr, Phe). Its elevated levels were previously found in people with insulin resistance and pre-diabetes^33^. The GSG ratio contains amino acids involved in glutathione synthesis—the glutamine divided by the sum of serine and glycine.

Tryptophan catabolism consists of three main pathways: kynurenine, serotonin, and indole^34^. In the presented study, whereas tryptophan itself was significantly decreased in the IHD group, the kynurenine and serotonin pathways were significantly increased. The kynurenine pathway (KP) represents the major degradation route of tryptophan catabolism. Recently, plenty of studies indicated an association of the KP with the progression of CVD, which may be explained by its pathogenetic involvement in cardiovascular risk factors, including hypertension, diabetes mellitus, dyslipidemia, and obesity, as well as in vascular inflammation and atherosclerosis^35^. The presented study identified significant increased levels of three intermediates of KP—anthranilic acid, kynurenic acid, and xanthurenic acid.

Serotonin was significantly increased. Serotonin is a potent vasoconstrictor and enhances the hypertensive effects of several vasoconstrictors, such as angiotensin and endothelin^36^. In the previous studies, serotonin was found to be increased in patients with primary hypertension and certain types of secondary hypertension^37,38^.

In contrast to increased serotonin level, we found that intermediates of the indole tryptophan catabolic pathway, consisting of indole-3-propionic acid, indole-3-butyric acid, and indole-3-carboxaldehyde, were decreased in IHD patients. These metabolites are presumably generated through the gut microbiota's direct or indirect metabolism^39,40^.

**Figure 5:**
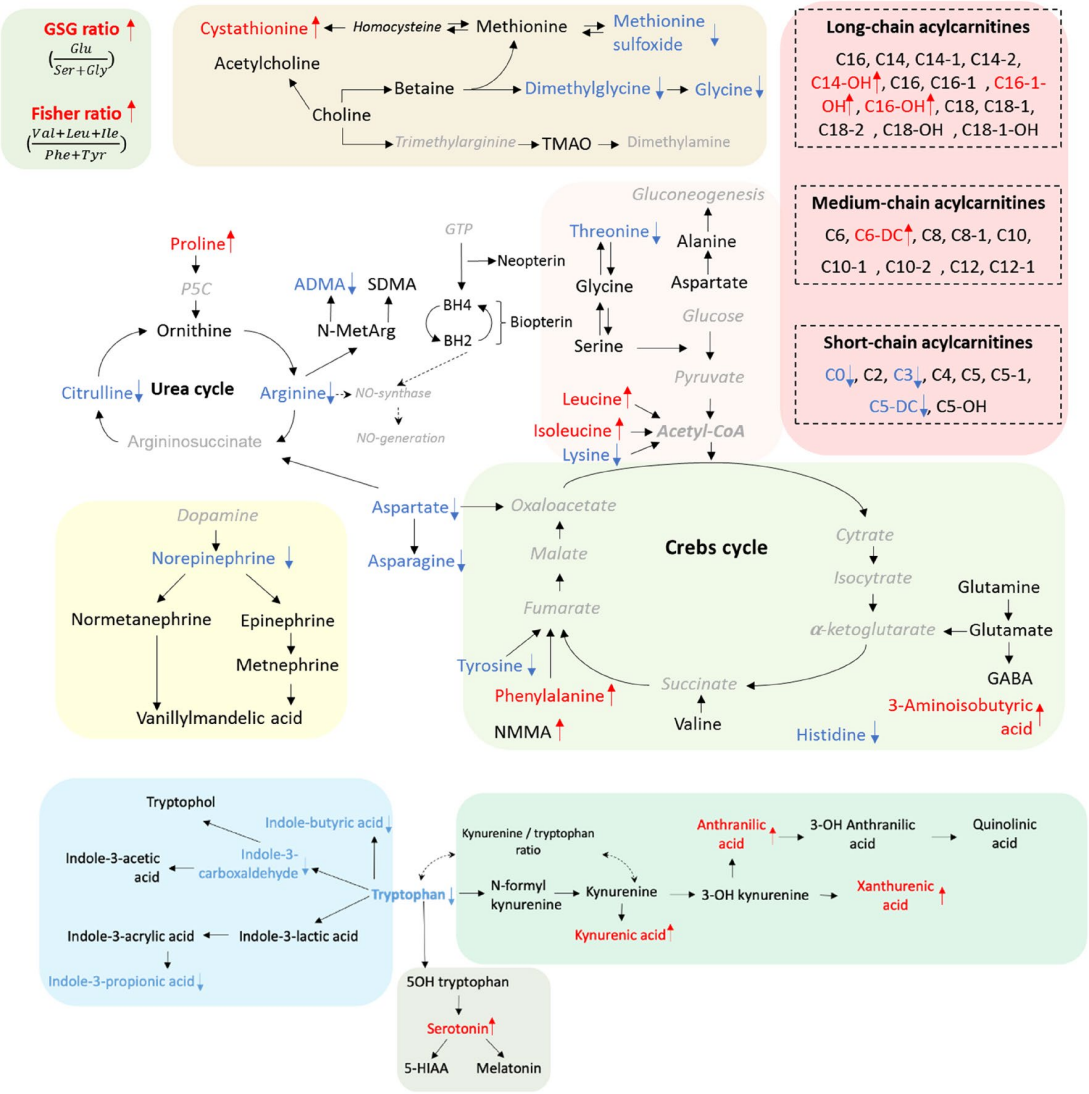
Significantly changed metabolites and metabolic pathways in the IHD pathogenesis and diagnostics.

Figure 5 summarizes the scheme of the significantly altered metabolic pathways associated with IHD.

### ML model

The introduction of machine learning methods to clinical diagnostics represents a promising healthcare approach. In the presented study, to find out the best model for IHD diagnostics, we compared five supervised ML algorithms, among which the best diagnostic accuracy was shown by the random forest model with an AUC value equal to 0.98.

**Figure 6:**
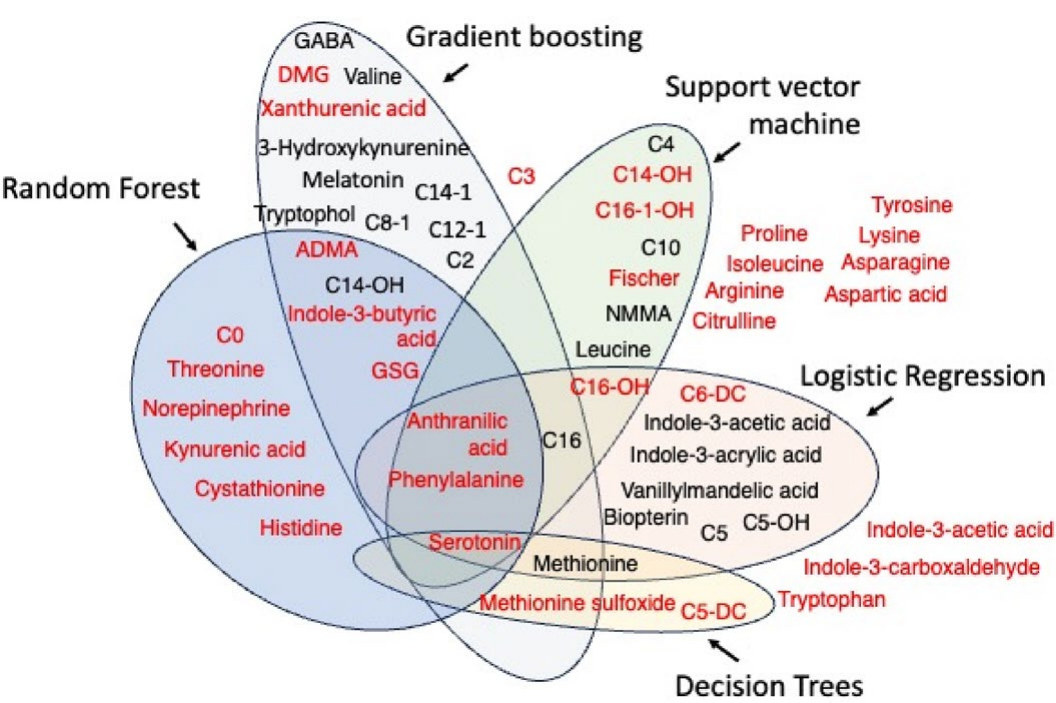
Plot of the features selected from the applied ML models. Metabolites marked in red had Mann–Whitney p-value < 0.05 and AUC < 0.65.

However, it should be mentioned that all applied algorithms except for the decision trees model provided slightly the same prediction quality. In this regard, we analyzed and compared the utilized in each model set of metabolites to elucidate those whose concentration level provided the highest impact on the diagnostics of IHD patients (Table S3). Figure 6 represents features utilized in each ML method, having p-value < 0.05 and AUC score > 0.65.

Based on this finding, we may conclude that metabolites Norepinephrine, Xanthurenic acid, Anthranilic acid, Serotonin, C6-DC, C14-OH, C16, C16-OH, GSG, Phenylalanine, and Methionine were found significant in most of the ML models. So, each of the ML model (RF; GB; SVH; LR) can be used separately as the preliminary diagnostic panel in patients with IHD. We hypothesize that these metabolites and ML model can be used for screening of IHD.

### Advantages and limitations of the study

The main advantage of the study is that the presented approach provides new insights into the development of IHD from the metabolic point of view and the selected metabolic panel may be applied in the diagnostics of IHD in clinical practice.

Limitations of this study must be addressed. We acknowledge that a larger cohort studies are recommended which would confirm the presented findings. At the same time, we identified unexpected changes in concentration levels of several endogenous metabolites in IHD patients’ compared to non-CVD subjects, that were previously unknown or disagreed with already published data.

## Conclusion

In conclusion, the presented study has successfully applied plasma metabolite-based ML modeling in screening IHD patients from non-CVD subjects, showing its efficacy in diagnostics of IHD with high levels of accuracy. Thus, even though this study was pilot, the presented results may facilitate future combination of ML-modeling and clinical metabolomics profiling for up-to-date diagnostics. Moreover, the suggested regression method for age-adjustment correction of metabolic data may be helpful in future metabolic studies with cohorts of non-balanced on-age participants. In addition, the identified, through the univariate analysis, significantly changed metabolites may also serve for the interpret of the molecular pathogenesis of IHD.

### Supplementary Information

Supplementary Information.

## Data Availability

All data generated and analysed during this study are included in this published article and its Supplementary Information files.
