# Supplementary material for: A novel preliminary metabolomic panel for IHD diagnostics and pathogenesis
Source: Sci Rep. 2024 Feb 1;14:2651. doi: 10.1038/s41598-024-53215-9 (PMC10834974; doi:10.1038/s41598-024-53215-9)
Supplement: Supplementary file 1 — Supplementary Information. [file 41598_2024_53215_MOESM1_ESM.docx]

**Supplementary material**

**A novel preliminary metabolomic panel for IHD diagnostics and pathogenesis**

Markin S.S.^1*^, Ponomarenko E.A.^1^, Romashova Yu. A.^1^, Pleshakova T.O.^1^, Ivanov S.V.^1^, Bedretdinov F.N.^1^, Konstantinov S.L.^2^, Nizov A.A.^3^, Koledinskii A.G.^4^, Girivenko A.I.^3^, Shestakova K.M.^6^, Markin P.A.^6^, Moskaleva N.E.^7^, Kozhevnikova M.V.^5^, Chefranova Zh.Yu.^2^, Appolonova S.A.^6,8^

Table S1. Coefficients of the regression models for adjustment of the metabolites by age

| **Metabolites** | **k** | **b** |
| --- | --- | --- |
| 3-Aminoisobutyric acid | 5.8e-03 | 0.84 |
| 3-Hydroxyanthranilic acid | 4.2e-05 | 0.02 |
| 3-Hydroxykynurenine | 9.6e-03 | 0.47 |
| GABA | 2.4e-04 | 0.12 |
| 5-Hydroxytryptophan | 1.9e-05 | 0.005 |
| Acetylcholine | 2.3e-05 | 0.0006 |
| Anthanilic acid | 1.9e-05 | 0.006 |
| Aspartic acid | 1.4e-04 | 0.17 |
| Biopterin | 6.1e-07 | 0.0009 |
| 5-Hydroxyindoleacetic acid | 3.2e-04 | 0.026 |
| Indole-3-acetic acid | 1.7e-03 | 1.89 |
| Indole-3-acrylic acid | -5.4e-04 | 0.21 |
| Indole-3-butyric acid | 8.6e-05 | 0.012 |
| Indole-3-carboxaldehyde | -3.4e-04 | 0.20 |
| Indole-3-lactic acid | -3.8e-04 | 0.74 |
| Indole-3-propionic acid | 1.2e-03 | 0.69 |
| Kynurenic acid | 3.0e-04 | 0.028 |
| Kynurenine | 4.7e-03 | 0.61 |
| Melatonin | -2.6e-06 | 0.0003 |
| Metanephrine | -1.9e-06 | 0.0003 |
| Neopterin | 7.0e-05 | 0.0037 |
| Norepinephirne | 1.6e-04 | 0.0076 |
| Normetanephrine | 1.6e-05 | 0.0013 |
| Quinolinic acid | 3.7e-03 | 0.178 |
| Serotonin | -1.6e-04 | 0.032 |
| Tryptophan | -1.3e-02 | 56.6 |
| Tryptophol | 1.6e-05 | 0.000036 |
| Vanillylmandelic acid | 1.3e-04 | 0.023 |
| Xanthurenic acid | -6.8e-05 | 0.009 |
| ADMA | 1.1e-02 | 0.37 |
| Betaine | 4.2e-01 | 27.1 |
| Choline | 1.9e-01 | 8.67 |
| Citrulline | 2.5e-01 | 13.91 |
| Cystathionine | 9.7e-04 | 0.025 |
| DMG | 1.7e-02 | 1.34 |
| Methionine | 9.5e-02 | 10.63 |
| Methionine sulfoxide | 9.9e-03 | 0.16 |
| NMMA | 6.6e-04 | 0.025 |
| Ornithine | 4.2e-01 | 28.28 |
| SDMA | 1.1e-02 | 0.63 |
| TMAO | 6.7e-02 | 1.11 |
| Alanine | 3.6e+00 | 213.77 |
| Arginine | 6.4e-01 | 52.04 |
| Asparagine | -7.9e-03 | 21.77 |
| Glutamine | 1.0e+00 | 39.24 |
| Glycine | 1.0e+00 | 188.13 |
| Histidine | 4.4e-01 | 38.03 |
| Isoleucine | 1.5e-01 | 68.18 |
| Leucine | 2.9e-01 | 91.45 |
| Lysine | 1.6e+00 | 145.65 |
| Methionine | 0.1e-01 | 20.047 |
| Phenylalanine | 9.3e-02 | 65.64 |
| Proline | 3.66 | 234.35 |
| Serine | 0.23 | 39.59 |
| Threonine | 0.87 | 114.68 |
| Tyrosine | 0.52 | 41.42 |
| Valine | 0.64 | 132.2 |
| C0 | 0.84 | 8.19 |
| C2 | 5.0e-01 | 13.20 |
| C3 | 6.8e-03 | 0.48 |
| C4 | 1.6e-03 | 0.075 |
| C5 | 1.5e-03 | 0.0036 |
| C5-1 | 7.9e-05 | 0.0066 |
| C5-DC | 1.6e-03 | 0.079 |
| C5-OH | -3.47e-05 | 0.010 |
| C6 | 8.0e-04 | 0.035 |
| C6-DC | 2.2e-05 | 0.0023 |
| C8 | 1.5e-03 | 0.15 |
| C8-1 | 1.17e-04 | 0.022 |
| C10 | 2.8e-04 | 0.24 |
| C10-1 | 1.47e-03 | 0.13 |
| C10-2 | 9.6e-05 | 0.0029 |
| C12 | 2.17e-04 | 0.044 |
| C12-1 | 6.17e-04 | 0.064 |
| C14 | 4.3e-05 | 0.027 |
| C14-1 | 6.6e-04 | 0.069 |
| C14-2 | 8.2e-05 | 0.061 |
| C14-OH | 1.7e-05 | 0.0013 |
| C16 | 8.67e-05 | 0.15 |
| C16-1 | 3.9e-04 | 0.017 |
| C16-1-OH | -4.1e-06 | 0.0036 |
| C16-OH | -4.87e-05 | 0.013 |
| C18 | 2.3e-04 | 0.059 |
| C18-1 | 1.2e-03 | 0.13 |
| C18-1-OH | 3.1e-05 | 0.0016 |
| C18-2 | 2.8e-04 | 0.099 |
| C18-OH | 2.1e-05 | 0.0018 |
| Kyn/Trp | 1.07e-04 | 0.0084 |
| AOR | -3.1e-03 | 1.89 |
| GABR | -1.1e-03 | 1.17 |
| Fischer ratio | -3.0e-03 | 2.68 |
| GSG ratio | 2.0e-03 | 0.19 |

Table S2. Tuned hyper parameters of the built ML model

| ML algorithm | Tuned hyperparameters |
| --- | --- |
| Logistic regression | C=1, penalty='l1', solver='liblinear' |
| SVM | probability=True, gamma=0.01, C=10 |
| Decision trees | criterion='gini', min_samples_leaf=5, min_samples_split=3, random_state=42 |
| Random Forest | criterion='entropy', max_depth=8, max_features=15 |
| Gradient boosting | learning_rate=0.5, max_depth=15, max_features='log2', min_samples_leaf=4, n_estimators=30, subsample = 0.6 |

Table S3. Metabolites selected as important in each built ML model and those with p-value < 0.05 and AUC > 0.65

|  | **RF** | **LR** | **SVM** | **GB** | **DT** | **P-value < 0.05** |
| --- | --- | --- | --- | --- | --- | --- |
| ADMA | + |  |  | + |  |  |
| Arginine |  |  |  |  |  | + |
| Citrulline |  |  |  |  |  | + |
| Normetanephrine |  |  | + |  |  |  |
| Cystathionine |  |  | + |  |  |  |
| Cystathionine | + |  |  |  |  | + |
| **Norepinephirne** | **+** |  |  |  | **+** | **+** |
| Vanillylmandelic acid |  | + | + |  |  |  |
| Biopterin |  | + | + |  |  |  |
| DMG |  |  |  | + |  | + |
| Methionine sulfoxide |  |  |  |  | + | + |
| Quinolinic acid |  |  | + |  |  |  |
| **Xanthurenic acid** |  |  | **+** | **+** |  | **+** |
| **Anthanilic acid** | **+** | **+** | **+** | **+** |  | + |
| Indole-3-butyric acid | + |  |  |  |  | + |
| Kynurenic acid | + |  |  |  |  | + |
| **Serotonin** | **+** | **+** | **+** | **+** | **+** | **+** |
| Indole-3-acrylic acid |  | + |  |  |  |  |
| Indole-3-acetic acid |  | + |  |  |  |  |
| tryptophan |  |  |  | + |  | + |
| 3-hydroxykynurenine |  |  |  | + |  |  |
| Melatonine |  |  |  | + |  |  |
| Indole-3-carboxaldehyde |  |  |  |  |  | + |
| Indole-3-propionic acid |  |  |  |  |  | + |
| C0 | + |  |  |  |  | + |
| C2 |  |  |  | + |  |  |
| C3 |  |  |  |  |  | + |
| C4 |  |  | + |  |  |  |
| C5 |  | + | + |  |  |  |
| C5-DC |  |  |  |  | + | + |
| C5-OH |  | + | + |  |  |  |
| **C6-DC** |  | **+** | **+** |  |  | **+** |
| C8-1 |  |  |  | + |  |  |
| C10 |  |  | + |  |  |  |
| C12-1 |  |  |  | + |  |  |
| **C14-OH** | **+** |  |  | **+** |  | **+** |
| C14-1 |  |  |  | + |  |  |
| C14-OH |  |  | + |  |  |  |
| **C16** |  | **+** | **+** | **+** |  |  |
| **C16-OH** | **+** | **+** | **+** |  |  | + |
| C16-1-OH |  |  | + |  |  | + |
| Lysine |  |  | + |  |  |  |
| Arginine |  |  | + |  |  |  |
| Valine |  |  |  | + |  |  |
| **GSG** | **+** |  |  | **+** |  | **+** |
| Histidine | + |  |  |  |  | + |
| **Phenylalanine** | **+** | **+** |  | **+** |  | **+** |
| Threonine | + |  |  |  |  | + |
| **Methionine** |  | **+** |  | **+** | **+** |  |
| GABA |  |  |  | + |  |  |
| Leucine |  |  | + |  |  | + |
| Fischer |  |  | + |  |  | + |
| NMMA |  |  | + |  |  |  |
| tyrosine |  |  |  |  |  | + |
| 3-Aminoisobutyric acid |  |  |  |  |  | + |
| Aspartic acid |  |  |  |  |  | + |
| Asparagine |  |  |  |  |  | + |
| Glycine |  |  |  |  |  | + |
| Lysine |  |  |  |  |  | + |
| Porline |  |  |  |  |  | + |
| Isoleucine |  |  |  |  |  | + |

*
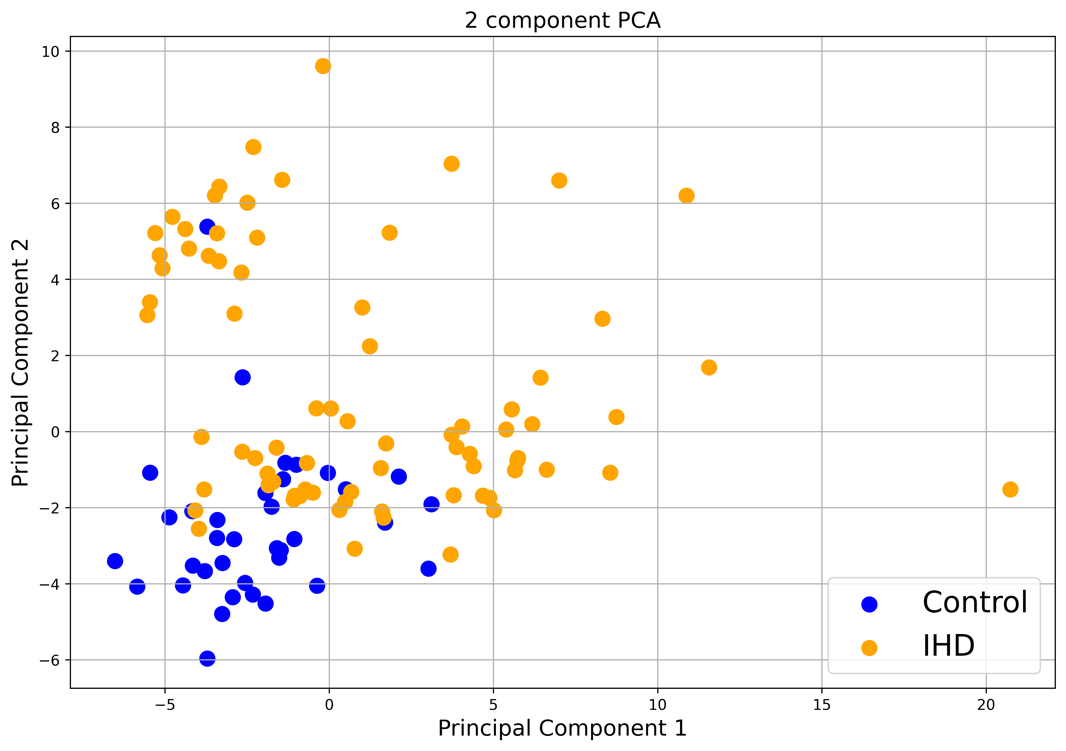
*

Figure S1. PCA analysis of the analyzed samples
